# Death from the Use of Chloroform

**Published:** 1883-06-20

**Authors:** Hunter McGuire

**Affiliations:** Ex-President Medical Society of Virginia, formerly Professor of Surgery Medical College of Virginia, etc., Richmond


### DEATH FROM USE OF CHLOROFORM.

By HUNTER McGUIRE, M. D.,
**Ex-President Medical Society of Virginia, formerly Professor of Surgery Medical College of Virginia, etc., Richmond.**

Mrs. M., æt. about 35, mother of six children, came to me October 6, 1882, with rupture of the perineum, the laceration extending into the rectum. She was about the usual height, rather thin in flesh, but healthy, and had never had any serious attack of sickness. Examination of heart and lungs revealed no sign of disease, nor was there any indication of disease of any of the other organs of the body.

On the 10th of October, the day appointed for the operation, she ate a simple breakfast; her bowels were moved by an aperient taken the night before, and at 9 and 11 o'clock she took five grains of quinine. At 2 o'clock, when I entered the room, I found her quite cheerful, and I thought with less than the usual apprehension about the operation and the use of chloroform. She was in bed, in her loosely-fitting night-clothes, her head supported by one pillow. About one drachm of Scquibb's chloroform was poured into a napkin, folded into the shape of a cone, open at both ends, and the lady began to inhale it. To avoid the danger of giving the vapor in too concentrated a form, the napkin at first was held several inches from the face, and gradually brought closer to her mouth and nose. She breathed the chloroform quietly, without choking, coughing or other resistance. Her face was turned towards me as I sat at the side of the bed, and she kept her eyes wide open, looking up into my face; the back of her right hand and arm lay across my knee, and I kept my finger on the pulse in this arm, noticing it carefully. Her wrist was thin, the artery prominent, and the pulsation distinct. I noticed at the time how clear, distinct and rather full the pulse was. As the day was warm, a window near the bed and a door opposite were opened. In the room with the patient and myself was Mrs. Jenkins, superintendent of the "Retreat." Outside of the open door, in the hall, were two assistants, Drs. Hugh Taylor and Lewis Wheat.

After she had breathed the anæsthetic for about two minutes and was still conscious, the pupils of both eyes slowly dilated to two or three times their natural size. When I saw this, I spoke to her, and she answered me intelligently. While she was speaking, the pulse in the arm upon which I had my finger stopped suddenly. It did not flutter and gradually fail, but abruptly ceased. The last stroke was as full and distinct as those which preceded it. A blow upon the heart would not have stopped it more abruptly, so sudden and complete was the *cardiac paralysis*, and this took place before she was under the influence of the chloroform and while she was yet conscious. When the heart stopped the face became palid, some convulsive movements of the muscles of the face and neck occurred, spasmodic, not tetanic in character; dilatation of the pupils slightly increased, and respiration continued for some seconds after the pulse ceased beating at the wrist. At least twenty-five or thirty respirations occurred after the cardiac paralysis, but the breathing was irregular, convulsive and imperfect. Fifteen or twenty seeonds intervened between the first appearance of dilatation of the pupils and arrest of the heart's action.

Nitrite of amyl, galvanism (both agents were close at hand), inversion of the body, and artificial respiration, kept up for an hour, were employed, but were of no avail.— *Va. Med. Monthly.*

